# Psychometric properties of the Suicidal Ideation Attributes Scale (SIDAS) in a longitudinal sample of people experiencing non-affective psychosis

**DOI:** 10.1186/s12888-021-03639-8

**Published:** 2021-12-16

**Authors:** Kamelia Harris, Gillian Haddock, Sarah Peters, Patricia Gooding

**Affiliations:** 1 Greater Manchester Mental Health NHS Foundation Trust, Manchester, UK; 2 Division of Psychology and Mental Health, School of Health Sciences, University of Manchester, Zochonis Building, Brunswick Street, Manchester, M13 9PL UK; 3 Manchester Academic Health Science Centre, MAHSC, Manchester, UK; 4 Manchester Centre for Health Psychology, University of Manchester, Manchester, UK

**Keywords:** SIDAS, Reliability, Validity, Psychometric properties, Longitudinal study

## Abstract

**Background:**

Suicidal ideation is a key precursor for suicide attempts and suicide deaths. Performing routine screening of suicide precursors can help identify people who are at high risk of death by suicide. This is, arguably, an important suicide prevention effort. The aim of this study was to assess the validity, reliability, and factor structure of the Suicidal Ideation Attributes Scale (SIDAS) in a three-month longitudinal study with people with a diagnosis of schizophrenia or non-affective psychosis and experiences of suicidal ideation and/or behaviours. It was predicted that the SIDAS would have high internal consistency, test-retest reliability, convergent, discriminant and construct validity.

**Methods:**

Ninety-nine participants experiencing psychosis completed the SIDAS at baseline and 89 participants completed it 3 months later. Additionally, participants completed a demographic questionnaire, the Beck Scale for Suicide Ideation, the Beck Hopelessness Scale, and the Defeat and Entrapment Scales. The internal consistency, test-retest reliability, convergent validity, and discriminant validity of the SIDAS were investigated in comparison to other constructs. Factor analysis was performed to examine the factor structure of the scale.

**Results:**

Principal component analysis yielded a theoretically coherent one-dimensional factor structure of SIDAS, suggesting good construct validity (PCA = .71). The SIDAS had high internal consistency (*α* = .89) and good test-retest reliability (*α* = .73). It was highly correlated with other self-report measures, including the Beck Scale for Suicide Ideation, Beck Hopelessness Scale, Defeat and Entrapment scales, indicating excellent construct validity.

**Conclusion:**

The SIDAS is a valid and reliable self-report instrument for assessing the severity of suicidal ideation in a population of people with a diagnosis of schizophrenia or non-affective psychosis. Further research should test the psychometric properties of the scale in individuals experiencing different mental health problems in cross-cultural settings, in order to establish its broader validity, reliability, and clinical utility.

## Introduction

It is estimated that approximately 800,000 people worldwide die by suicide every year [1]. In the UK, there were 6507 suicide deaths and, in the USA, there were 48,344 suicide deaths in 2018 [2, 3]. Suicide deaths are associated with substantial societal and economic costs, amounting to £1.7 million per suicide annually in England and $69 billion overall in the USA [4, 5]. In addition, suicide-related experiences, such as suicidal ideation, attempts, plans, and urges can have a tremendous negative psychological impact on individuals and the people around them [6, 7]. Assessing suicide risk is challenging in research and mental health care settings due to the complex and multifaceted nature of suicide-related experiences across different contexts [6, 8, 9]. Furthermore, detailed individual assessments of suicidal experiences may be unfeasible as time, organisational and financial resources are limited in mental health services [10–12]. Therefore, measuring the risk of suicidal ideation, plans, attempts and deaths accurately, using robust, concise measures that are sensitive to change, is of major importance in clinical practice [13–16].

The Suicidal Ideation Attributes Scale (SIDAS) [16] is a relatively new, brief scale, designed to measure the severity of suicidal ideation in the past month. It includes five items assessing the frequency, controllability and likelihood of suicide attempts related to suicidal ideation, as well as the associated distress and interference with daily activities with which suicidal experiences are associated (see Table 1 for the scale items).

**Table 1:** SIDAS items

Item	Description
1	In the past month, how often have you had thoughts about suicide?
2	In the past month, how much control have you had over these thoughts?
3	In the past month, how close have you come to making a suicide attempt?
4	In the past month, to what extent have you felt tormented by thoughts about suicide?
5	In the past month, how much have thoughts about suicide interfered with your ability to carry out daily activities, such as work, household tasks or social activities?

According to a comprehensive review of self-report scales for suicidal ideation and behaviours, the SIDAS, in addition to two other measures (i.e., Depressive Symptom Index Suicidality Subscale (DSI-SS), Suicidal Behaviors Questionnaire-Revised (SBQ-R)), showed the greatest potential for use in research [17]. To this date, the scale has been validated in an online Australian adult sample [16], in a French sample of adolescents and adults [18], and a Chinese sample of university students [19]. The SIDAS was reported to have high convergent validity with the Columbia Suicide Severity Scale (C-SSRS) [20] in the adult Australian sample [16]. The test-retest reliability and convergent validity of the SIDAS with scales measuring related constructs, such as hopelessness, defeat and entrapment, as well as other established suicidal ideation scales, such as the Beck Scale for Suicide Ideation [21], have not been investigated. Hopelessness, defeat and entrapment were selected due to their associations with increased levels of suicidal ideation and behaviours [22–24]. The longitudinal design of the current study assessed the test-retest reliability and the broader psychometric properties of the SIDAS.

Rating scales should be validated in the population in which they are being used [15]. The psychometric properties of the SIDAS have not been investigated in a clinical sample of people with a diagnosis of schizophrenia or non-affective psychosis. Information regarding the generalisability and clinical utility of the SIDAS is potentially useful to mental health professionals in the assessment of suicidal ideation severity, particularly in people with non-affective psychosis because suicidal ideation, acts and deaths are highly prevalent in this population [25–27]. This study had two aims:

1. To assess the factor structure of the SIDAS. It was predicted that the original one-factor structure of SIDAS would be replicated, as reported in the study by van Spijker et al. [16].
2. To examine the internal consistency, test-retest reliability, convergent validity and discriminant validity of the SIDAS, compared to established scales measuring suicidal ideation (i.e., the Beck Scale for Suicide Ideation), suicide-related constructs (i.e., the Beck Hopelessness Scale [28], the Defeat and Entrapment scales [29]), and non-suicide-related constructs (i.e., the Connor-Davidson Resilience Scale [30]). It was predicted that the SIDAS would have high (i.e., >.8 [31]) internal consistency, test-retest reliability, convergent, discriminant and construct validity, thus illustrating the reliability and validity of this measure in people with severe mental health problems.

## Methods

### Design

A longitudinal design was used with a three-month follow-up period. Ethical approval was granted by the North West-Greater Manchester Central Research Ethics Committee (18/NW/0181).

### Participants

Participants were recruited from UK National Health Service (NHS) mental health trusts as part of a study about resilience to suicidal ideation and behaviours [32]. An additional 19 participants in the current study sample were recruited upon completion of a clinical trial investigating the effectiveness of a psychological talking therapy for people who experience or have experienced psychosis and suicidal ideation and/or feelings (i.e., Cognitive AppRoaches to coMbatting Suicidality (CARMS) [33]; ISRCTN: 17776666). Information leaflets were displayed in public areas within the trusts, so that individuals were able to self-refer to the study by contacting the first author (KH). Mental health professionals could refer potential participants, based on the eligibility criteria provided by KH, during mental health team meetings. Potential participants were given a participant information sheet and had at least 24 h to decide whether they wished to participate. If they were interested, KH arranged a time and place, convenient to them, to meet and complete the measures. Assessments were conducted after obtaining participants’ informed consent. Participants were contacted 3 months later and asked to complete the same measures.

This study included individuals with experiences of: i. current non-affective psychosis (e.g., hallucinations, delusions) or a schizophrenia diagnosis (including schizophrenia, schizophreniform disorder, schizoaffective disorder, delusional disorder or psychotic disorders not otherwise specified), confirmed by a member of their mental health care team, ii. lifetime suicidal ideation, urges, plans and/or acts, iii. 18 years or older, iv. English-speaking, and v. having capacity to provide informed consent. People experiencing affective psychosis (e.g., bipolar disorder, major depressive disorder, mood disorder), dementia or organic brain disorder were not eligible to participate in the current study.

### Measures

#### Suicidal Ideation Attributes Scale (SIDAS) [16]

The SIDAS is a five-item scale measuring the frequency and severity of suicidal ideation in the past month. Each item is scored on a scale from 0 (Never) to 10 (Always). Items include: “In the past month, how often have you had thoughts about suicide?” and “In the past month, how close have you come to making a suicide attempt?” (see Table 1). Scores ≥21 indicate a high risk of suicidal behaviour. The scale has been reported to have a high internal consistency in a large Australian general population sample (*α* = .91) [16]. Cronbach’s alpha in the current study was .89.

#### Beck Scale for Suicide Ideation (BSS) [21]

The Beck Scale for Suicide Ideation is a 21-item scale assessing suicidal ideation, planning and intent in the past week. For each item, respondents can choose between three options, for example, “I have no wish to die”, “I have a weak wish to die” or “I have a moderate to strong wish to die”. Research with participants with psychosis reported the scale to have a high internal consistency (*α* = .96) [34]. Cronbach’s alpha in the current study was .94.

#### Beck Hopelessness Scale (BHS) [28]

The Beck Hopelessness Scale is a 20-item scale designed to assess the presence of hopeless thoughts and beliefs in the past week. Example items include: “My future seems dark to me,” to which respondents can respond with “True” or “False”. Scores > 14 indicate severe hopelessness. The scale had a high internal consistency in a study with people with recent onset schizophrenia (*α* = .93) [35]. Cronbach’s alpha in the current study was .92.

#### Defeat and Entrapment Scales [29]

These scales contain 16 items each, measuring feelings of failed struggle and low social rank (e.g., “I feel that I am one of life’s losers”) over the past week for the Defeat scale, and being trapped and having the desire to escape (e.g., “I am in a situation I feel trapped in”) without a specified time frame for the Entrapment scale. Both are measured on a five-point scale. The Defeat scale ranges from “Never” to “Always/all the time”, whereas the Entrapment scale ranges from “Not at all like me” to “Extremely like me”. The internal consistency for the Defeat scale ranged between .93 and .94, and for the Entrapment scale between .86 and .93 in a sample of people experiencing psychosis [23]. Cronbach’s alpha in the current study was .91 and .93 for the Defeat and Entrapment scales, respectively.

#### Connor-Davidson Resilience Scale (CD-RISC) [30]

The CD-RISC contains 25 items, rated on a five-point scale (0-not true at all; 1-rarely true; 2-sometimes true; 3-often true; 4-true nearly all of the time) which measure key resilience components, namely, perceived ability to thrive, bounce back and adapt to change following adversity. Example items include: “I can deal with whatever comes” and “I tend to bounce back after illness or hardship”. The scale has been used in samples of people with a range of mental health problems, including schizophrenia, anxiety, depression, and PTSD [36–39]. The scale had a high internal consistency in a study with people recruited from the community, primary care settings, community mental health settings, and clinical trials with people experiencing PTSD and generalised anxiety disorder (*α* = .87) [30]. Cronbach’s alpha in the current study was .93.

### Analysis

To test for normality, the *z*-scores for the skewness and kurtosis of the variables were calculated for which skewness and kurtosis scores were divided by their standard errors. If the obtained value is greater than ±1.96, this indicates that data are non-normally distributed. This method is recommended for studies with small sample sizes [40]. Depending on the distribution of the data, Pearson’s correlation coefficient (for normally distributed data) or Spearman’s rho (for non-normally distributed data) were used to examine associations between variables.

Exploratory factor analysis using the principal component method [41] was performed to assess whether the SIDAS items measured a single construct, in accord with van Spijker et al.’s analysis [16]. Exploratory factor analysis was used because the factor structure of the SIDAS has not been tested with people experiencing severe mental health problems, specifically, schizophrenia or non-affective psychosis. The analysis was performed both on the entire sample (*n* = 99) and on a sub-sample of participants who reported suicidal ideation at baseline (i.e., a score greater than zero; *n* = 54).

Paired samples *t*-tests were used to assess statistical differences between baseline and follow-up scores for all measures. Independent samples t-tests were used to assess the statistical differences between the SIDAS means reported in the current study and van Spijker et al.’s study [16]. Probability values at the alpha level of .05 indicated significant results.

The internal consistency (i.e., Cronbach’s alpha coefficient) and convergent validity of the SIDAS were assessed and compared to those of the Beck Scale for Suicide Ideation [21], the Beck Hopelessness Scale [28], and the Defeat and Entrapment scales [29]. Correlations between the SIDAS and the Beck Scale for Suicide Ideation, Beck Hopelessness Scale, the Defeat and Entrapment Scales were used to assess the convergent validity of the SIDAS in the current study sample. The discriminant validity [42] of the SIDAS was assessed and compared to a non-suicide measure, namely, the Connor-Davidson Resilience Scale.

For the interpretation of convergent validity, associations greater than .7 and .8 indicate acceptable or high convergence, respectively [31, 43]. Associations of less than .5 indicate poor internal consistency [31, 43]. Data analysis was carried out using SPSS version 25.0.

## Results

### Participant characteristics and baseline scores for key variables

Participants’ demographic characteristics are presented in Table 2. Most participants were white British, unemployed, single, and identified as male.

**Table 2:** Participant demographic characteristics at baseline and follow-up time points

Characteristic	Baseline mean (*SD*) / *n* (*n* = 100)	Follow-up mean (*SD*) / *n* (*n* = 90)	Range
Age (yrs)	41.07 (13.06); 100	41.30 (13.35); 89	19–75
Gender (male)	80; 100	75; 90	
Ethnicity			
White British	73; 100	67; 89	
Black British	9; 100	8; 89	
Mixed race	3; 100	3; 89	
South Asian	6; 100	5; 89	
North African	1; 100	1; 89	
White other	8; 100	5; 89	
Occupation			
Unemployed	81; 100	73; 90	
Employed	4; 100	3; 90	
Student	4; 100	3; 90	
Volunteer	3; 100	4; 90	
Retired	8; 100	7; 90	
Education			
Primary education	20; 100	15; 89	
Secondary education	33; 100	31; 89	
Higher education	47; 100	43; 89	
Relationship status			
Single	79; 100	68; 89	
Married	8; 100	8; 89	
In a relationship	7; 100	9; 89	
Divorced	5; 100	4; 89	
Unknown	1; 100	0; 89	
Living arrangements			
Alone	28; 100	26; 90	
With family	19; 100	17; 90	
With partner	8; 100	7; 90	
With friend	2; 100	2; 90	
Student accommodation	1; 100	1; 90	
Supported housing	11; 100	13; 90	
Rehabilitation unit	16; 100	14; 90	
Inpatient	15; 100	10; 90	
Case note diagnosis			
Schizophrenia	59; 100	52; 90	
Schizoaffective disorder	16; 100	15; 90	
Psychosis NOS	23; 100	21; 90	
Delusional disorder	2; 100	2; 90	
Self-reported antipsychotic medication			
First generation (typical)	12; 100	11; 89	
Second generation (atypical)	52; 100	46; 89	
Third generation (atypical)	13; 100	14; 89	
First (typical) & Second (atypical) generation	4; 100	2; 89	
First (typical) & Third (atypical) generation	5; 100	3; 89	
Second (atypical) & Third (atypical) generation	5; 100	5; 89	
None	4; 100	5; 89	
Unknown	5; 100	3; 89	
Duration of contact with mental health services (yrs)	15.55 (11.60); median = 14; 91		1–51

*Psychosis NOS* Psychosis Not Otherwise Specified

The baseline and follow-up means, standard deviations and medians for the SIDAS, Beck Scale for Suicide Ideation, Beck Hopelessness Scale, Defeat and Entrapment scales are presented in Table 3, in addition to *t*-test values examining differences in scores across these time points. The scores on the Beck Scale for Suicide Ideation and Entrapment were significantly lower at follow-up, compared to baseline assessment. There were no statistically significant differences between the remaining baseline and follow-up measures (i.e., SIDAS, Beck Hopelessness Scale, and Defeat).

**Table 3:** Descriptive statistics for the baseline and follow-up Suicidal Ideation Attributes Scale (SIDAS), Beck Scale for Suicide Ideation (BSS), Beck Hopelessness Scale (BHS), Defeat and Entrapment scales scores

Outcomes	Baseline mean (*SD*); median; *n*	Follow-up mean (*SD*); median; *n*	Baseline range	Follow-up range	*t* (*p*-value)	*Cohen’s d*
SIDAS	11.56 (13.18); 5; 99	10.67 (11.77); 7; 89	0–46	0–39	.69 (.50)	.09
BSS	8.63 (8.79); 4; 99	7.24 (8.03); 4; 89	0–36	0–33	**2.17 (.04)**	.23
BHS	9.00 (6.03); 9;100	8.43 (6.15); 7; 90	0–20	0–20	1.14 (.29)	.13
Defeat	32.14 (15.51); 31; 99	32.25 (14.40); 32; 89	0–64	0–61	−.19 (.84)	.02
Entrapment	29.92 (17.58); 31; 99	26.64 (16.16); 28; 89	0–73	0–64	**2.30 (.02)**	.24

*SIDAS* Suicidal Ideation Attributes Scale, *BSS* Beck Scale for Suicide Ideation, *BHS* Beck Hopelessness Scale. Bold values indicate significant results at the alpha level of .05

The *t*-test is assessing the statistical difference between baseline and follow-up scores. A score equal to, or greater than, 21 on the SIDAS indicates high risk of suicidal behaviour [16]. Twenty-nine participants in the current study had a SIDAS score greater than 21 at baseline. In comparison, 21 participants had a score greater than 21 at follow-up.

The baseline mean, standard deviation, median, mode and range of each item comprising the SIDAS scale are presented in Table 4. In the full sample of 99 people, the mean SIDAS scores for each item were 2.63 for frequency, 2.15 for controllability, 1.19 for closeness to attempt, 2.85 for distress, and 2.74 for interference with daily activities. In comparison, the scores in the sample reported in the study by van Spijker et al. [16] were 1.42 for frequency, 1.42 for controllability, 0.57 for closeness to attempt, 1.19 for distress, and .90 for interference with daily activities. The mean SIDAS scores were significantly higher in the current study, compared to van Spijker et al.’s study [16] (*t* (8) = 3.504, *p* < .05). The baseline SIDAS mean total score increased from 11.56 in the full sample (*n* = 99) to 21.19 in the sub-sample of people reporting suicidal ideation (*n* = 54). These figures were 5.5 in the full sample (*n* = 1352) and 13.3 in the sub-sample reporting suicidal ideation (*n* = 560) in the study of van Spijker et al. [16]

**Table 4:** Baseline mean, standard deviation, median, mode and range of each item comprising the Suicidal Ideation Attributes Scale (SIDAS)

SIDAS item	Mean (*SD*); *n*	Median	Mode	Range
1. Frequency	2.63 (3.24); 99	1	0	0–10
2. Controllability^a^	2.15 (3.10); 99	.00	0	0–10
3. Closeness to acting	1.19 (2.37); 99	.00	0	0–9
4. Distress	2.85 (3.44); 99	.00	0	0–10
5. Interference with daily activities	2.74 (3.27); 99	.00	0	0–10

^a^Controllability was reverse scored prior to analysis

### Normality tests

The tests of normality indicated that the baseline Beck Scale for Suicide Ideation, SIDAS and Defeat scales were non-normally distributed, whereas the Entrapment scale was normally distributed. These results were similar for the follow-up measures, except for Defeat and Entrapment which were normally distributed. As the majority of baseline and follow-up measures were non-normally distributed, Spearman’s rho correlation coefficients were used.

### Aim 1: to assess the factor structure of the SIDAS

The factor loadings for the entire sample and the sub-sample reporting suicidal ideation at baseline are presented in Table 5. In the full sample, the single factor had an eigenvalue of 3.57, accounting for 71.46% of the total variance. In the sub-sample reporting suicidal ideation at baseline, the single factor had an eigenvalue of 2.69, accounting for 53.85% of the total variance. All five items had factor loadings greater than .71 in the full sample and greater than .47 in the sub-sample reporting suicidal ideation at baseline. Therefore, this suggests the SIDAS represents a unidimensional construct which means that participants can be evaluated in a single, overall score of suicidal ideation.

**Table 5:** Factor analysis of the Suicidal Ideation Attributes Scale (SIDAS) for the full sample (*n* = 99) and sub-sample of participants reporting suicidal ideation at baseline (*n* = 54)

	Full baseline sample (*n* = 99)	Baseline suicidal ideation sub-sample (*n* = 54)
1. Frequency	.88	.72
2. Controllability^a^	.71	.47
3. Closeness to acting	.77	.71
4. Distress	.93	.86
5. Interference with daily activities	.92	.84
Eigenvalue	3.57	2.69
Variance accounted for	71.46%	53.85%

^a^Controllability was reverse scored prior to data analysis

### Aim 2: to assess the internal consistency, convergent validity, discriminant validity and test-retest reliability of the SIDAS

#### Internal consistency

Cronbach’s alpha coefficients indicated that all scales in this study had high internal consistency at baseline. The internal consistency of the SIDAS was .89, for the Beck Scale for Suicide Ideation it was .94, for the Beck Hopelessness Scale it was .92, and for the Defeat and Entrapment scales, it was .91 and .93 in this study, respectively.

#### Convergent validity

Correlations with self-report measures related to suicide, specifically, the Beck Scale for Suicide Ideation, Beck Hopelessness Scale, Defeat and Entrapment scales at baseline were calculated to determine the convergent validity of the SIDAS (see Table 6). There was a statistically significant relationship between the severity of suicidal ideation assessed by the SIDAS and the Beck Scale for Suicide Ideation (*r* = .72, *p* < .01) at baseline. In addition, greater severity of suicidal ideation assessed by the SIDAS was associated with higher levels of hopelessness (Beck Hopelessness Scale *r* = .52, *p* < .01), Defeat (*r* = .49, *p* < .01) and Entrapment (*r* = .52, *p* < .01) at baseline. A similar pattern of results was observed for the follow-up variables. Specifically, there were statistically significant relationships between the follow-up SIDAS and the Beck Scale for Suicide Ideation (*r* = .62, *p* < .01), the Beck Hopelessness Scale (*r* = .61, *p* < .01), the Defeat (*r* = .54, *p* < .01) and the Entrapment (*r* = .51, *p* < .01) scores.

**Table 6:** Spearman correlation coefficients between the Suicidal Ideation Attributes Scale (SIDAS), Beck Scale for Suicide Ideation (BSS), Beck Hopelessness Scale (BHS), Defeat and Entrapment Scales for the baseline and follow-up sample

	SIDAS	BSS	BHS	Defeat	Entrapment	SIDAS FU	BSS FU	BHS FU	Defeat FU	Entrapment FU
SIDAS	1	.719**	.518**	.493**	.516**	.734**	.582**	.524**	.431**	.394**
BSS	.719**	1	.600**	.509**	.483**	.644**	.675**	.583**	470**	.412**
BHS	.518**	.600**	1	.736**	.642**	.508**	.460**	.757**	.708**	.571**
Defeat	.493**	.509**	.736**	1	.782**	.513**	.390**	.557**	.745**	.586**
Entrapment	.516**	.483**	.642**	.782**	1	.472**	.313**	.557**	.622**	.685**
SIDAS FU	.734**	.644**	.508**	.513**	.472**	1	.623**	.611**	.543**	.507**
BSS FU	.582**	.675**	.460**	.390**	.313**	.623**	1	.547**	.508**	.382**
BHS FU	.524**	.583**	.757**	.557**	.557**	.611**	.547**	1	.755**	.683**
Defeat FU	.431**	.470**	.708**	.745**	.622**	.543**	.508**	.755**	1	.767**
Entrapment FU	.394**	.412**	.571**	.586**	.685**	.507**	.382**	.683**	.767**	1

*SIDAS* Suicidal Ideation Attributes Scale, *BSS* Beck Scale for Suicide Ideation, *BHS* Beck Hopelessness Scale, *FU* Follow-up

^**^p < .001

#### Discriminant validity

A measure of resilience which was the Connor-Davidson Resilience Scale was used to examine the discriminant validity of the SIDAS. The Spearman’s rho correlation coefficient (*r* = −.39, *p* < .01) indicated that the two measures were negatively correlated and, therefore, appeared to be measuring divergent constructs. In comparison, the Spearman’s rho correlation coefficient for the relationship between the Connor-Davidson Resilience Scale and the Beck Scale for Suicide Ideation was similar (*r* = −.44, *p* <. 001), indicating that the two measures were negatively correlated.

#### Test-retest reliability

This analysis was significant and showed evidence for good test-retest reliability of baseline and follow-up SIDAS scores over 3 months (*r* = .73, *p* < .001; see Table 6).

## Discussion

There were three key, and novel, findings of the current study. First, the SIDAS was a reliable and valid measure that accurately identified experiences of suicidal ideation in people with a diagnosis of schizophrenia or non-affective psychosis when compared with established measures, such as the Beck Scale for Suicide Ideation, the Beck Hopelessness Scale and the Defeat and Entrapment scales, and the Connor-Davidson Resilience Scale. Second, this study showed that the SIDAS can be administered in a UK sample of people experiencing a severe mental health problem, namely, non-affective psychosis. This adds to the work demonstrating the importance of the SIDAS in the Australian general population [16]. That said, it must be noted that the SIDAS has not been evaluated in minority cultures such as in people from Black and Asian ethnic minorities, and different religious backgrounds. Third, the SIDAS had high test-retest reliability over a period of 3 months.

The mean scores on the Beck Scale for Suicide Ideation, Beck Hopelessness Scale, and the Defeat and Entrapment scales reflect those reported in previous studies including community samples of people with a diagnosis of schizophrenia [34, 44]. For example, three studies have reported mean Beck Scale for Suicide Ideation scores between 4.91 and 5.64 [23, 34, 44]. The mean Beck Scale for Suicide Ideation score in this study was 8.63. Two of these studies have reported mean Beck Hopeless Scale scores between 7.21 and 7.41 [23, 44]. The mean Beck Hopelessness Scale score in this study was 9.00. One of these studies reported mean Defeat and Entrapment scores of 28.56 and 24.67, respectively [23]. In comparison, the participants in this study reported Defeat and Entrapment scores of 32.14 and 29.92, respectively. Of note, a small proportion of the current sample in this study (i.e., 31%) was recruited from inpatient and recovery settings which may have resulted in marginally elevated scores on these measures, compared to those reported in previous studies including community clinical samples.

There was a difference in the total variance accounted for between the full study sample and the sub-sample of people experiencing suicidal ideation at baseline. This could be attributed to the significantly lower baseline SIDAS scores in the full sample.

The SIDAS is a relatively brief measure of suicidal ideation severity and may have omitted factors that are relevant to experiences of suicidal ideation. For example, other issues, such as duration of suicidal ideation may contribute to more severe suicide-related experiences, such as suicide plans and suicide attempts [20]. In addition, factors such as hopelessness, desire to live, and burdensomeness have been associated with suicidal experiences but are not included in the SIDAS [45–47]. This study showed that in a sample of people with non-affective psychosis, the SIDAS moderately correlated with measures of related constructs, such as hopelessness, defeat and entrapment, and negatively correlated with a measure of a non-suicide-related construct, namely, psychological resilience. It should be noted that some of the correlations between these constructs were low (e.g., SIDAS and defeat *r* = .49). This could be a result of a potential conceptual overlap between defeat, entrapment and hopelessness as evidenced by previous studies [48–50].

Both the SIDAS and the Beck Scale for Suicide Ideation showed almost identical high reliability and validity, indicating good clinical utility. However, this is the first study assessing the psychometric properties of the SIDAS in a sample of people with a severe mental health problem, that is, non-affective psychosis. Further research is needed to thoroughly evaluate the clinical significance and clinical utility (i.e., the extent to which the use of the scale is associated with potentially preventing suicide-related outcomes) [51] of the SIDAS in people experiencing different mental health problems (e.g., bipolar disorder, depression, anxiety, personality disorders) before it can be implemented as a measure of suicidal ideation severity in clinical practice. Furthermore, future research would profit from investigating whether any SIDAS items, in particular, are more or less predictive of suicidal behaviours, such as urges and attempts.

### Limitations

This study has two limitations worth noting. First, the participant sample in this study was recruited from a project examining the relationships between psychological resilience and suicidal ideation and behaviours in people experiencing non-affective psychosis which included a predetermined set of scales. Consequently, this may limit the scope of the research questions and the potential findings as data collection had already been completed prior to analysis. For example, relevant factors that are associated with increased severity of suicidal experiences, such as previous suicide attempts, instances of self-harm or number of hospitalisations in relation to mental health problems could not be examined in the current study. Future studies could include these factors in the assessment of the psychometric properties of the SIDAS. Second, the predictive validity of the SIDAS has not been established in clinical samples. For instance, the relationsip between baseline suicidal ideation measured by the SIDAS and future suicide attempt severity, frequency and/or suicide death has not been examined. Therefore, the validity of SIDAS scores in predicting suicide-related behavioural outcomes in clinical samples prospectively represents a key area for future research.

Two points need to be considered in relation to the predictive validity of the SIDAS. First, it has been shown that suicidal ideation can fluctuate considerably [52, 53]. The SIDAS measures the severity of suicidal ideation in the last month, therefore, it is a useful retrospective measure of these experiences. It would be beneficial to test the validity of the SIDAS as a contextual, momentary assessment of suicidal ideation severity in an experience sampling framework [54]. Second, due to the innacuracy of measures in predicting suicidal behaviours and suicide deaths, the National Institute for Health and Care Excellence [55] recommended a shift from risk assessment to a needs assessment approach in the allocation of care. That said, suicide scales remain a useful means for individual needs assessment [56].

## Conclusion

The SIDAS is a reliable and valid, continuous self-report measure of the severity of suicidal ideation in people with a diagnosis of schizophrenia or non-affective psychosis. This study showed that the SIDAS can be administered in a UK sample of people with severe mental health problems, which complements work with this measure in a different culture (e.g., Australian general population [16]). Further research should test the psychometric properties of the SIDAS cross-culturally, in people experiencing different mental health problems, and using a longer-term follow-up period, in order to establish its validity, reliability and clinical utility.

## Data Availability

The datasets used and/or analysed during the current study are available from the corresponding author on reasonable request.

## Abbreviations

SIDAS: Suicidal Ideation Attributes Scale
BSS: Beck Scale for Suicide Ideation
BHS: Beck Hopelessness Scale
FU: Follow Up
